# Testing the Genomic Shock Hypothesis Using Transposable Element Expression in Yeast Hybrids

**DOI:** 10.3389/ffunb.2021.729264

**Published:** 2021-08-23

**Authors:** Marika Drouin, Mathieu Hénault, Johan Hallin, Christian R. Landry

**Affiliations:** ^1^Institut de Biologie Intégrative et des Systèmes, Université Laval, Québec, QC, Canada; ^2^Département de Biochimie, de Microbiologie et de Bio-Informatique, Université Laval, Québec, QC, Canada; ^3^PROTEO - Regroupement Québécois de Recherche sur la Fonction, l'Ingénierie et les Applications des Protéines, Québec, QC, Canada; ^4^Centre de Recherche en Données Massives de l'Université Laval, Université Laval, Québec, QC, Canada; ^5^Département de Biologie, Université Laval, Québec, QC, Canada

**Keywords:** transposable element, hybridization, genomic shock, differential expression analysis, RNA sequencing, retrotransposon, yeast

## Abstract

Transposable element (TE) insertions are a source of structural variation and can cause genetic instability and gene expression changes. A host can limit the spread of TEs with various repression mechanisms. Many examples of plant and animal interspecific hybrids show disrupted TE repression leading to TE propagation. Recent studies in yeast did not find any increase in transposition rate in hybrids. However, this does not rule out the possibility that the transcriptional or translational activity of TEs increases following hybridization because of a disruption of the host TE control mechanisms. Thus, whether total expression of a TE family is higher in hybrids than in their parental species remains to be examined. We leveraged publically available RNA-seq and ribosomal profiling data on yeast artificial hybrids of the *Saccharomyces* genus and performed differential expression analysis of their LTR retrotransposons (Ty elements). Our analyses of total mRNA levels show that Ty elements are generally not differentially expressed in hybrids, even when the hybrids are exposed to a low temperature stress condition. Overall, only 2/26 Ty families show significantly higher expression in the *S. cerevisiae* × *S. uvarum* hybrids while there are 3/26 showing significantly lower expression in the *S. cerevisiae* x *S. paradoxus* hybrids. Our analysis of ribosome profiling data of *S. cerevisiae* × *S. paradoxus* hybrids shows similar translation efficiency of Ty in both parents and hybrids, except for Ty1_cer showing higher translation efficiency. Overall, our results do not support the hypothesis that hybridization could act as a systematic trigger of TE expression in yeast and suggest that the impact of hybridization on TE activity is strain and TE specific.

## Introduction

Genome diversification resulting from interspecific hybridization allows the development of novel phenotypes, creating an adaptive potential that can empower hybrids to colonize new niches (Runemark et al., 2019; Steensels et al., 2021). However, combining divergent genomes can also lead to hybrid sterility or inviability caused by genetic incompatibilities (Muller, 1939; Maheshwari and Barbash, 2011). Other consequences of hybridization include genetic instability that can affect hybrid fitness and hybrid genome evolution. One example is an increased rate of proliferation of transposable elements (TEs) in hybrid genomes (McClintock, 1984).

TEs are dispersed repeated sequences that can propagate within a genome (Bourque et al., 2018). They are ubiquitous in eukaryotes but represent various genome proportions in different species. For instance, they represent from 3% of the yeast *Saccharomyces cerevisiae* genome and up to 80% of some plant genomes (Meyers et al., 2001; Carr et al., 2012; Platt et al., 2018). TE abundance explains much of the variation in genome size among eukaryotes (Elliott and Gregory, 2015). TEs generate genomic insertions that are mostly selectively neutral or slightly deleterious (Doolittle and Sapienza, 1980). Because of their detrimental effects, TEs are often compared to parasites spreading selfishly within their host genome (Orgel and Crick, 1980). However, beneficial consequences of TE insertions have also been reported (González and Petrov, 2009; Arkhipova, 2018). One source of deleteriousness of TE multiplication is the resulting increase in genome size and complexity, which can lead to increased instability and thus to phenotypic changes (Kidwell and Lisch, 1997). Instability can come from ectopic genome recombination that can cause chromosomal instability and structural variants, such as translocations and inversions (Luning Prak and Kazazian, 2000). TEs can also affect gene expression by disrupting existing regulatory sequences at insertion sites or by providing new ones (Niu et al., 2019). For instance, some TEs contain promoters and enhancers that regulate their own expression and that can affect expression of neighboring genes (Naito et al., 2009; Lisch, 2013; Chuong et al., 2017). Hence, TE mobilization is likely to reduce the fitness of the host.

Hosts have TE repression mechanisms that limit the spread of TEs and that vary depending on the organism and the type of element (Saha et al., 2015; Sigman and Slotkin, 2016; Luo and Lu, 2017). However, the ability to regulate TEs could be disrupted in certain environmental or genomic conditions, including in hybrids (Horváth et al., 2017). According to the genomic shock hypothesis proposed by McClintock in 1984, stress and regulatory interference triggered by interspecific hybridization could lead to TE mobilization (McClintock, 1984). One possible cause of such interference could be the antagonistic coevolution between TEs and host repression. The merger of two divergent genomes brings together two TE populations that may differ in terms of composition and relative abundance, and which may form co-adapted units with their respective host genome. Hybridization may thus perturb these coevolved units, leading to a disruption of transposition regulation mechanisms and TE mobilization. Many studies comparing TE transcription level in parental species and their hybrids found an overexpression of some TEs in hybrids (Kelleher et al., 2012; Dion-Côté et al., 2014; Renaut et al., 2014; Lopez-Maestre et al., 2017), whereas others found no overexpression in hybrids for the majority of the TEs (Josefsson et al., 2006; Goebel et al., 2018). Thus, McClintock's hypothesis has been both confirmed and refuted depending on the system, so the phenomenon of genomic shock resulting from hybridization is not universal.

One of the model systems harboring active TEs but in which TE activity has not been found to increase in hybrids is the budding yeast. Two recent studies examined whether hybridization causes an increase in the transposition rate of TEs in yeast hybrids and did not find evidence for it (Hénault et al., 2020; Smukowski Heil et al., 2021). The relative simplicity of the yeast genome, along with extensive knowledge of the life cycle of its TEs, makes it an ideal system to yield deeper insights into the regulation of transposition in hybrids. Yeast TEs are long terminal repeat (LTR) retrotransposons also called Ty elements, which replicate via a copy-and-paste mechanism including an RNA intermediate. Five main Ty families are found in the model species *S. cerevisiae*: Ty1, Ty2, Ty3, Ty4, and Ty5 (Kim et al., 1998; Carr et al., 2012). A larger diversity of TE families are found in other *Saccharomyces* species (Bleykasten-Grosshans and Neuvéglise, 2011). LTR retrotransposons comprise an internal coding sequence flanked by two LTRs in direct orientation. While active families have full-length copies, the vast majority of genomic insertions are non-coding solo LTR elements remaining from LTR-LTR recombination of full-length elements (Kim et al., 1998). The abundance of solo LTR and full-length Ty sequences varies depending on Ty families and strains (Bleykasten-Grosshans et al., 2013). Much knowledge on the biology of Ty elements (and LTR retrotransposons in general) stems from studies of the Ty1 and Ty3 families from *S. cerevisiae* (Curcio et al., 2015; Sandmeyer et al., 2015). The life cycle of the Ty elements is analogous to that of retroviruses (Rowley, 2017; Czaja et al., 2020). Ty elements are transcribed into mRNAs encoding two overlapping open reading frames (ORFs), *GAG*, and *POL*, which are, respectively, translated in the Gag and Gag-Pol proteins. Gag is a structural protein, while Gag-Pol is a catalytic protein containing protease, integrase, and reverse transcriptase domains. These Ty-encoded components are necessary for the reverse-transcription of the mRNA into cDNA and for the genomic integration of a new Ty copy, completing the transposition cycle.

The life cycle of Ty elements is complex, and measures of transposition are accordingly diverse. The recent investigations performed on yeast tested mobilization by measuring Ty copy number changes after experimental evolution (Hénault et al., 2020), or by experimentally measuring transposition rates with reporter assays (Smukowski Heil et al., 2021). Although these studies concluded in an absence of mobilization, they have not ruled out the possibility that TEs are transcriptionally derepressed, as seen for some animal and plant species (Kelleher et al., 2012; Dion-Côté et al., 2014; Renaut et al., 2014; Lopez-Maestre et al., 2017; Laporte et al., 2019). For instance, transcriptional derepression could be compensated by Ty regulation at the post-transcriptional or post-translational levels. Studying an intermediate stage of the Ty life cycle allows one to better understand the host mechanisms enabling or repressing transposons activity. Transcript abundance and intensity of translation are good proxies to evaluate the activity of these TEs in hybrids.

Our objective was to test if TEs are more expressed in F1 hybrids of yeast. We performed differential expression analysis on two publicly available RNA sequencing (RNA-seq) datasets of *Saccharomyces* species (*S. cerevisiae* × *S. uvarum* and *S. cerevisiae* × *S. paradoxus* diploid hybrids and their diploid parents). We also analyzed a published ribosome profiling dataset of *S. cerevisiae* × *S. paradoxus* hybrids to evaluate Ty translation efficiency. We tested whether the total expression of each active Ty family is higher in hybrids than in their parental species at the transcriptional and the translational level. Our analyses show that Ty elements are generally not differentially expressed in hybrids, even when the hybrids are exposed to a low temperature stress condition.

## Materials and Methods

### Dataset and Strain Description

We performed differential expression analysis using three RNA-seq datasets of diploid parental and lab constructed interspecific diploid hybrids of the *Saccharomyces* genus. The first dataset, which we will call DS1 throughout the remainder of the article, is from Schraiber and collaborators (Schraiber et al., 2013). DS1 includes two biological replicates of *S. uvarum* (CBS 7001) and *S. paradoxus* (CBS432) parents hybridized with *S. cerevisiae* (YHL068) and grown at 25°C. The second dataset (DS2) is from Hovhannisyan et al. (2020). DS2 includes three biological replicates of *S. cerevisiae* (YPS128), *S. uvarum* (UWOPS99-807.1.1) and their hybrids grown at two temperatures: 30°C and 12°C. The third dataset (DS3) is a ribosome profiling dataset that includes two replicates of hybrids of *S. cerevisiae* (S288C) and *S. paradoxus* (CBS432) and the matched total mRNA RNA-seq libraries (McManus et al., 2014). Sample description and accession numbers of DS1, DS2, and DS3 can be found in Supplementary Tables 1, 2). Raw RNA-seq data were retrieved from NCBI Sequence Read Archive (Leinonen et al., 2011) with SRA Toolkit version 2.10.8 with–split-files option https://trace.ncbi.nlm.nih.gov/Traces/sra/sra.cgi?cmd=show&f=software&m=software&s=software.

Downstream analysis was slightly different for the datasets because different sequencing methods were used to generate them. For DS1, the authors used 3′-end RNA-seq on samples with polyadenylated transcripts enriched twice. cDNAs were sequenced using paired-end (PE) 36 bp mode on an Illumina IIx Genome Analyzer (Yoon and Brem, 2010). For DS2, libraries were sequenced in PE 50 bp (30°C) and PE 75 bp (12°C) mode on an Illumina HiSeq 2500 platform after a single poly-A mRNA selection. These data are also reverse stranded. For DS3, stranded mRNA libraries were prepared by poly-A enrichment and random fragmentation before sequencing in single-end 50 bp mode on an Illumina HiSeq 2000 instrument.

### Quality Control and Data Filtering

For DS1 and DS2, we used FastQC v0.11.8 https://www.bioinformatics.babraham.ac.uk/projects/fastqc/ and MultiQC v1.9 (Ewels et al., 2016) for raw sequencing data quality control. For DS1, we used a custom script based on the ShortRead R package (Morgan et al., 2009) to only keep read pairs where one of the reads has two or more T nucleotides at the 5′ end. This way, we discarded reads that had no sign of mRNA poly-A tail. For DS2, no filtering was done because we confirmed that there were no poly-A tails in raw reads. For both DS1 and DS2, we used Trimmomatic v0.36 (Bolger et al., 2014) with Truseq3 adaptors and the following parameters: 2:30:10 LEADING:3 TRAILING:3 SLIDINGWINDOW:4:15 MINLEN:30 for adaptor trimming. For DS3, reads were trimmed for base quality and adapter sequences using fastp v0.21.0 (Chen et al., 2018) with options -l 20-5-3-r and a custom Illumina adapter database.

### TE Reference Sequences and Annotations

Our custom library of representative internal and LTR sequences of each Ty family inserted in the reference genomes includes Ty1, Ty2, Ty3, Ty4, and Ty5 from *S. cerevisiae*, Ty1, Ty3, and Ty5 from *S. paradoxus* and Tsu4 from *S. uvarum*. The library of internal and LTR reference sequences of Ty1-Ty5 in *S. cerevisiae* was retrieved from file S1 of Carr and collaborators (Carr et al., 2012). For the Tsu4_uva sequence, the only Ty family found in *S. uvarum*, we used the GenBank reference sequence under accession number AJ439550.1 (Neuvéglise et al., 2002). For the Ty1, Ty3, and Ty5 families in *S. paradoxus*, we generated representative reference sequences from the genome of the strain CBS432 (Yue et al., 2017) using custom Python v3.9.1 scripts (Van Rossum and Drake, 2009). Internal and LTR sequences were extracted from the whole-genome assembly using reference annotations (Yue et al., 2017). Multiple sequence alignments were produced using MUSCLE v3.8.31 (Edgar, 2004) and consensus sequences were derived using the BioPython package v1.76 (Cock et al., 2009) with a minimal frequency threshold of 0.5 and replacing ambiguous positions with Ns. BLASTN v2.7.0 (Camacho et al., 2009) was used to find the best hit (highest bit score) for each consensus among the annotated sequences, which was chosen as a reference. In the case of *S. cerevisiae* and *S. paradoxus*, the reference genomes from which the Ty reference sequences were retrieved in our study are often the exact same strains employed in the transcriptomics datasets.

Nucleotide divergence between *S. cerevisiae* and *S. paradoxus*, the two most closely related parental species of our datasets, is generally high enough to allow unambiguous short read mapping. To ensure this is the case for Ty families, we analyzed the sequence similarity profiles between the Ty1 and Ty3 families, which are orthologous between both species (namely Ty1_cer-Ty1_par and Ty3_cer-Ty3_par). We extracted all full-length annotated Ty1 and Ty3 sequences from the S288c and CBS432 genomes (Yue et al., 2017). We computed multiple sequence alignments for Ty1_cer-Ty1_par sequences and Ty3_cer-Ty3_par sequences using MUSCLE v3.8.31 (Edgar, 2004). Pairwise nucleotide identity between *S. cerevisiae* and *S. paradoxus* sequences was computed using custom Python v3.9.1 scripts (Van Rossum and Drake, 2009). Alignments were split into 100 bp non-overlapping windows. For each window, nucleotide identity between each pair of sequences was approximated by counting the number of mismatches, ignoring insertions and deletions. The average nucleotide identity was computed across all pairwise comparisons for a given window.

### Genome Sequences and Annotations

For DS2, whole genome assembly and annotations of *S. cerevisiae* YPS128 strain were downloaded in GenBank under accession code Bioproject PRJEB7245 (Yue et al., 2017). The reference genomes and annotations for the other strains in DS1 and DS2 were downloaded from www.saccharomycessensustricto.org (Scannell et al., 2011). For each species, we combined ultrascaffolds and unplaced regions in the same fasta file to get one complete reference genome, as described in Hovhannisyan et al. (2020). The annotation file format conversion was done with gffread v0.12.4 (Pertea and Pertea, 2020) and the code in GFF_GTF_conversion.txt file found at https://github.com/Gabaldonlab/Hybrid_project. For DS1 and DS2, we used RepeatMasker v.4.1.0. (Smit et al., 2015) with –gff and –lib 2 “custom TEs library” options to detect and mask interspersed repeats and low complexity DNA sequences by replacing them by Ns in the reference parental genome sequences. After masking Ty copies, we added our custom library of representative internal and LTR sequences of each Ty family to the fasta files and annotation files as if Ty sequences were additional chromosomes. Hybrid genomes were constructed by concatenating the corresponding masked parental genomes and adding reference sequences of Tys present in parental species. This was done so that each hybrid reference genome contains only a single copy of Ty from each family on which the RNA-seq reads could be aligned. We added the concatenated internal and LTR sequences to reference genomes and genome annotation for DS1, whereas we added our library of internal and LTR Ty sequences as separate fasta entries for DS2. Ty internal sequences were concatenated with their 3′ LTR in DS1 in order to select the read pairs including only one read mapping on the internal sequence and the other read mapping on the 3′ LTR along with read pairs mapping on the internal sequence. Since this dataset was generated with a 3′-end sequencing method, it is expected that the mapping should extend from the 3′ extremity of the Ty internal sequence to the end of the transcript, in the 3′ LTR.

For DS3, we generated a concatenated hybrid reference genome by merging chromosomes from *S. paradoxus* CBS432 and *S. cerevisiae* S288C reference genomes (Yue et al., 2017) and adding reference sequence for each Ty family that comprised the 5′ LTR and the complete internal sequence. Chromosomal sequences were hard-masked (replaced by Ns) for all Ty sequences using reference annotations and a custom Python script.

### RNA-seq Mapping

Alignments of RNA-seq reads of hybrids and their parents were performed on concatenated masked parental genomes supplemented with Ty reference sequences. For DS1, we used Bowtie2 v2.3.4.1 (Langmead and Salzberg, 2012) with–local option to do a soft clipped alignment with reads having poly-T at 5′ end, as described in Schraiber et al. (2013). To exclude spurious transcription from solo LTRs, we kept only pairs for which the first read mapped inside an internal sequence using samtools view with -f 67 -h options and the coordinates of internal sequences on the pseudo-chromosomes. Lists of unique read IDs were extracted from the resulting bam files. These lists allowed us to retrieve the corresponding pairs using the FilterSamReads tool from picard-tools v2.23.2 https://broadinstitute.github.io/picard/ with option FILTER=includeReadList. Bam files of Ty elements and of all host chromosomes were combined with GatherBamFiles of picard-tools. For DS2, we performed RNA-Seq read mapping with the splice-junction aware mapper STAR (Dobin et al., 2013) with default parameters, as described in Hovhannisyan et al. (2020). For DS3, reads were mapped on the concatenated references using HISAT2 v2.2.1 (Kim et al., 2019). Secondary alignments were filtered out using samtools view v1.9 (Li et al., 2009) with option -F256.

### Differential Expression Analysis

For genes and TE total expression quantification of DS1 and DS2, we used the Rsubread featureCounts R function (Liao et al., 2014) to assign mapped reads to genomic features and generate raw read count matrices of Ty. We used featureCounts with minFragLength=5, GTF.attrType=“transcript_id” options for the non-stranded DS1. For the reverse-stranded DS2, we used countMultiMappingReads=TRUE, fraction=TRUE, strandSpecific=2, minFragLength=5, GTF.attrType=“transcript_id.”

We normalized data of raw read count matrices with the median of ratios normalisation method implemented in DESeq2 (Love et al., 2014). This normalization accounts for sequencing depth and RNA composition using the negative binomial distribution. To evaluate if TE expression differs between hybrid and parental genomes, we performed differential expression analysis with DESeq2 (Love et al., 2014), which uses Wald test for significance testing. For both datasets, we compared Ty expression from their internal sequences in hybrids with the ones in the parent having full-length elements. Parental species were set as the first level in the treatment factor and only the subgenomes of these parental species (including their corresponding Ty sequences) were kept for DESeq2 analysis of each correspondant hybrid and parental samples. For DS2, we performed a multi-factor analysis with design = counts ~ temperature + species + species:temperature. Since the temperature effect was only significant for the Ty1 family, we normalized and analysed Ty1 expression data at 30 and 12°C separately. To identify Ty families differentially expressed in hybrids, we used an adjusted *p*-value value threshold of 0.05.

For DS3, analyses were conducted with scripts from the Plastid Python library (Dunn and Weissman, 2016). Since reference transcript annotations included no untranslated regions (UTRs), gene annotations were modified to add a 50 bp padding upstream of each CDS to simulate 5′ UTRs, allowing to capture 5′ offsets of ribosome protected fragments (RPF) reads. For RPF libraries, 5′ offsets were estimated using the psite script from Plastid (Dunn and Weissman, 2016) to yield the position of ribosomal P-sites for each read length. The phase_by_size script was used to determine which read lengths yielded optimal phasing. We restricted read lengths to the 27–32 bp range inclusively. Read counts per position and per gene were computed using the get_count_vectors and counts_in_region scripts, respectively, from Plastid (Dunn and Weissman, 2016), with variable 5′ offsets. For total mRNA libraries, read counts per gene were computed using the counts_in_region script from Plastid (Dunn and Weissman, 2016) with the central position of each gene. Counts were analyzed in DESeq2 (Love et al., 2014) by fitting the model counts ~ species + experiment + species:experiment, where the species categorical variable includes the hybrid and one of its parental species, and the experiment categorical variable comprises total mRNA and RPF. The latter interaction term corresponds to translation efficiency. Two independant models of this class were run for sets of genes/Ty and samples corresponding to each parental subgenome. Similarly, we ran a separate class of DESeq analyses, but restricting to mRNA libraries and using the model counts ~ species. For all models, log2 fold change values were corrected with the default apeglm shrinking method (Zhu et al., 2019).

### Coverage Depth Uniformity

For DS1 and DS2, we generated heatmaps of RNA-seq coverage depth along Ty sequences by generating bed files with samtools depth -a to get per-base coverage depth values along Ty sequences. For DS3, the output of the get_count_vectors script from Plastid was used to get position-wise coverage depth values (Dunn and Weissman, 2016). Custom Python scripts were used to compute z-scores of mean coverage depth in 75 bp-wide non-overlapping windows. For the three datasets, we also calculated Transcripts Per Million (TPM) for each sample.

### Code

All bash, R and Python code used in this study can be found at https://github.com/Landrylab/Drouin_et_al_2021.

## Results

**Figure 1:**
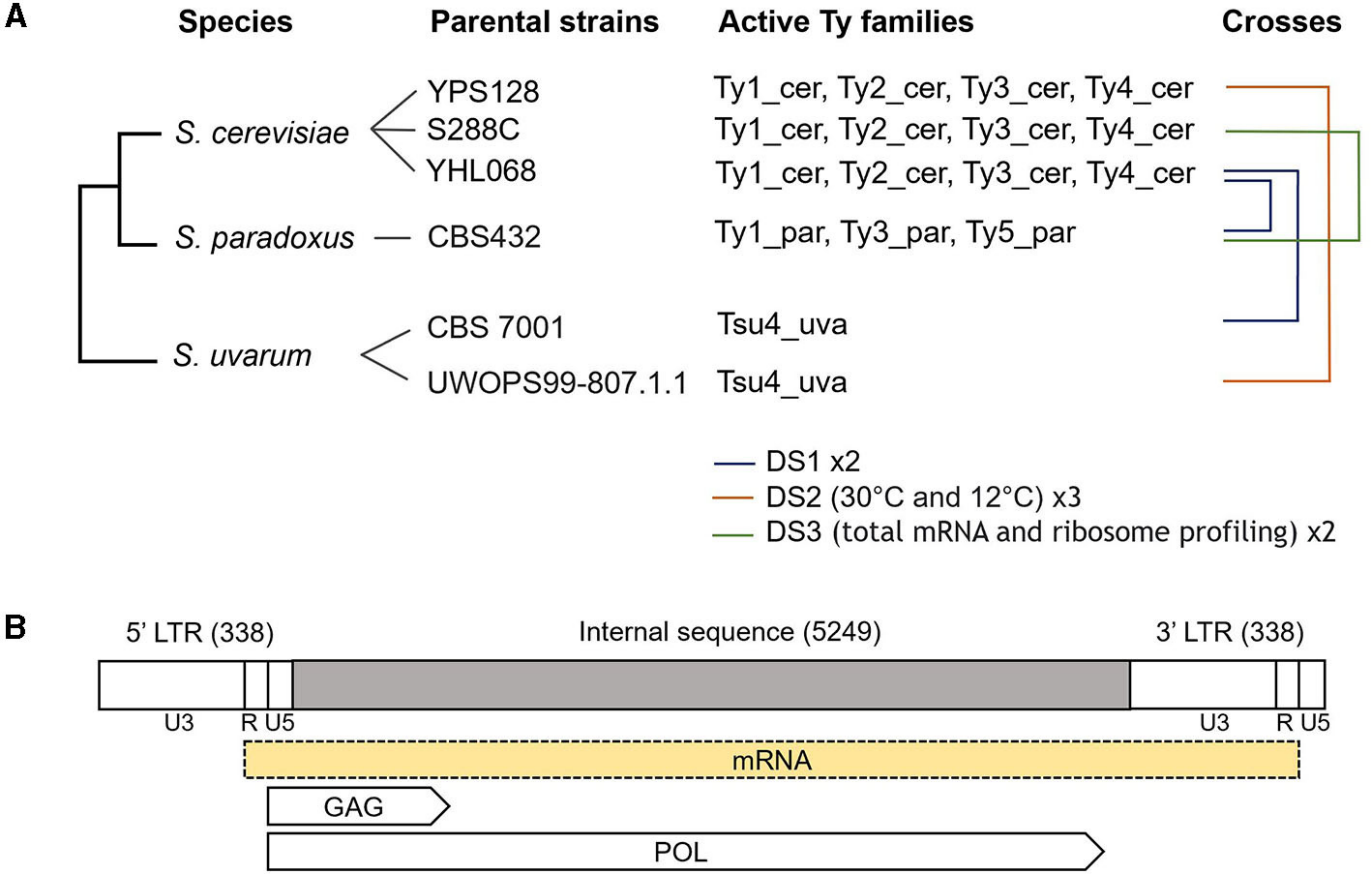
**(A)** Experimental design of the crosses performed in the three expression datasets. The phylogenetic tree (not to scale) details parental species and strains used, as well as their Ty family content. Active families are defined as comprising at least one full-length element, with a complete internal sequence and two flanking LTRs. To distinguish Ty families that are orthologous across parental species, family names are complemented with their host species (Ty1_cer corresponding to Ty1 from *S. cerevisiae*). Brackets show the crosses from DS1 (Schraiber et al., 2013), DS2 (Hovhannisyan et al., 2020), and DS3 (McManus et al., 2014). Number of samples per cross and specific conditions of the datasets are indicated in the legend. **(B)** Annotations of a Ty1 full-length sequence (not to scale). The length of the internal and LTR sequences are indicated between parentheses. The *GAG* and *POL* overlapping open reading frames (ORFs) are represented by arrows delimited by their respective start and stop codons. The *GAG* start codon is 40 bp upstream of the end of the 5′ LTR and the *POL* stop codon is 25 bp upstream of the beginning of the 3′ LTR. Ty1's canonical transcript, which is represented by a yellow box, starts in the 5′ LTR (including the R and U5 regions) and ends near the end of the 3′ LTR (including the U3 and R regions). The figure is inspired from the Figure 5 of Curcio et al. (2015).

In order to determine if TEs are derepressed in newly formed hybrids, either at the transcription or the translation level, we performed differential expression analysis on three publicly available expression datasets: DS1, DS2 and DS3 (Schraiber et al., 2013; McManus et al., 2014; Hovhannisyan et al., 2020). We tested whether the expression of each active Ty family is different in hybrids compared to their parental species. These datasets include *S. cerevisiae x S. uvarum* (Sc x Su) and *S. cerevisiae x S. paradoxus* (Sc × Sp) hybrids, as well as different growth temperatures (30 and 12°C) (Figure 1A).

### Coverage Along Ty Sequences

**Figure 2:**
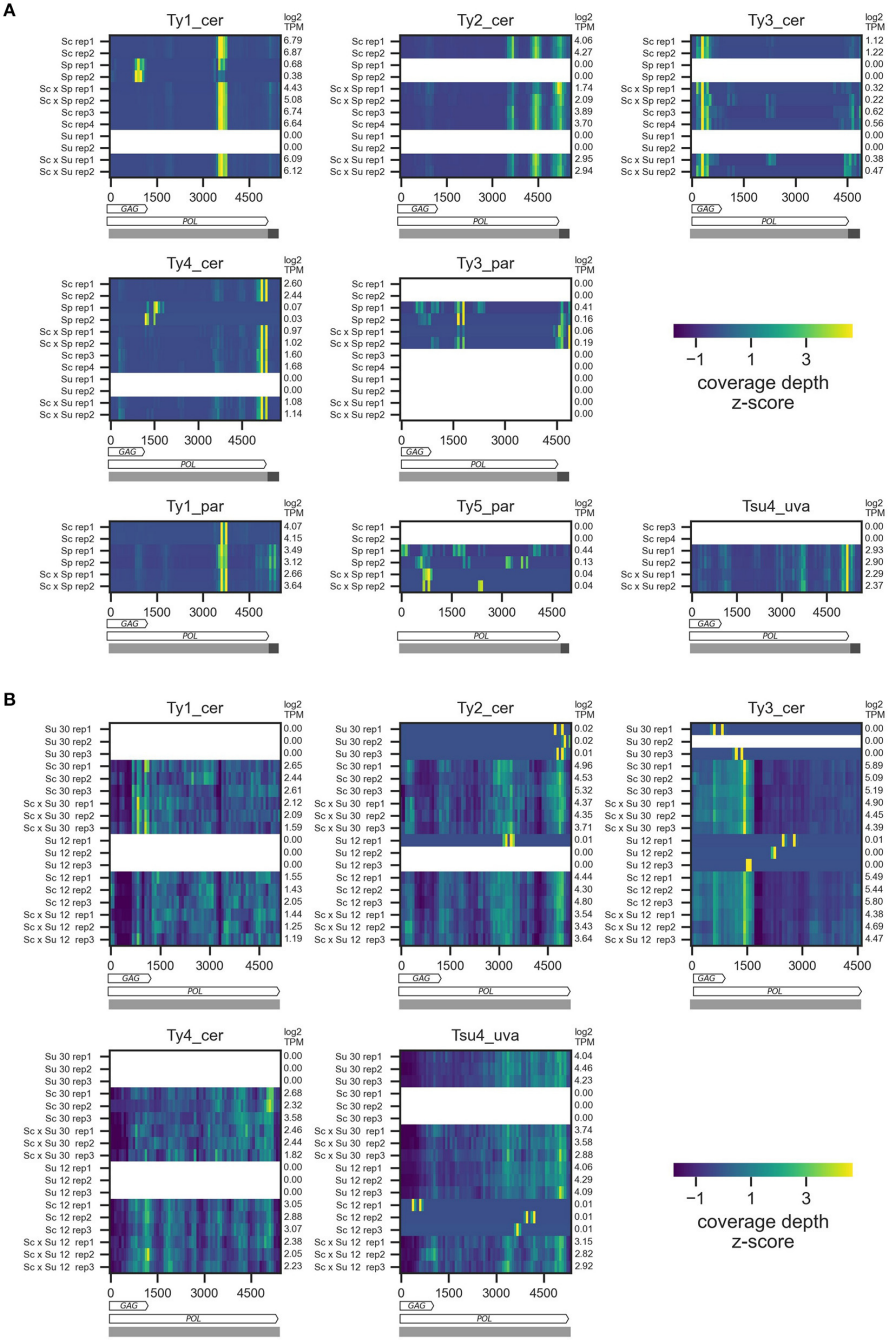
RNA sequencing coverage depth along Ty reference sequences for DS1 **(A)** and DS2 **(B)**. Z-scores of mean coverage depth are shown for each sample by 75 bp-wide non-overlapping bins along Ty reference sequences. In **(A)**, rows show the two replicates for each parental species and hybrids. In **(B)**, rows show the three replicates for each parental species and hybrids at the two temperatures. For each Ty family, crosses for which one of the parents is known to harbor this family are shown. As Ty3_par was included in the Ty analysis of *S. cerevisiae* in Carr et al. (2012), we also investigated its content in Sc × Su samples. Transcripts Per Million (TPM) of each sample are indicated at the right of the heatmaps. Horizontal bars below the heatmaps represent the annotation of the reference sequences used for read mapping, with internal sequences and LTRs shown, respectively, in light and dark grey. The *POL* and *GAG* (except for Ty5_par) ORFs are shown as arrows.

To confirm that alignments of RNA-seq reads on Ty sequences were comparable between parental and hybrid samples, we first examined the coverage along Ty sequences (Figure 1B). For DS1, coverage is concentrated in narrow peaks along the sequences (Figure 2A). The distributions of peaks differ between Ty families and contrast the expectation from the 3′ end RNA-seq, in which coverage should be limited to the 3′ portion of transcripts (Tandonnet and Torres, 2017; Ma et al., 2019). Since we select for A nucleotides at the 3′ end of RNA-seq reads, homopolymers of A in the reference sequences could be associated with higher mapping, for instance because of library contamination by fragments that would not correspond to the 3′ portion of transcripts. However, we generally observe no poly-A stretches that could explain the high coverage in these regions. This suggests that major alternative polyadenylation sites may exist in Ty internal sequences. Nevertheless, these distributions are consistent within Ty families, thus allowing us to compare hybrid and parental samples. For DS2 and DS3, the coverage is fairly uniform along the sequence, as expected from mRNA sequencing with a single poly-A selection (Figure 2B, Supplementary Figure 1). Both replicates of parents and hybrids also have similar coverage profiles between the two temperatures in DS2. Furthermore, for all datasets, some parental species known to be devoid of full-length elements for a given family exhibit low and irregular coverage, consistent with non-specific mapping. These results suggest that RNA-seq data can be harnessed for differential expression analysis of Ty elements between hybrids and parental samples, and between environmental conditions.

One exception is the Ty1 family of *S. cerevisiae* and *S. paradoxus*, which shows evidence of substantial non-specific mapping (i.e., *S. paradoxus* reads mapping on Ty1_cer and vice-versa; Figure 2, Supplementary Figure 1). Some of this non-specific mapping is due to the close sequence similarity of the Ty1 GAG region between the two species (Supplementary Figure 2), consistent with interspecific introgression for this locus (Czaja et al., 2020). We thus excluded the GAG region from the analysis of Ty1 in Sc × Sp hybrids. Nevertheless, a substantial fraction of non-specific mapping occurred outside of GAG. Using libraries of the parental species, we estimated that between 5 and 15% of Ty1 reads of a given species map on the reference sequence of the cognate species. While this non-specific mapping biases both Ty1_cer and Ty1_par expression levels, the latter has at least five times lower expression levels across library types (Figure 2; Supplementary Figure 1) and, as such, is disproportionately more affected by non-specific mapping coming from highly abundant *S. cerevisiae* reads. As a consequence, we excluded Ty1_par from our analyses.

### Most Ty Show no Differential Expression Between Parents and Hybrids

**Figure 3:**
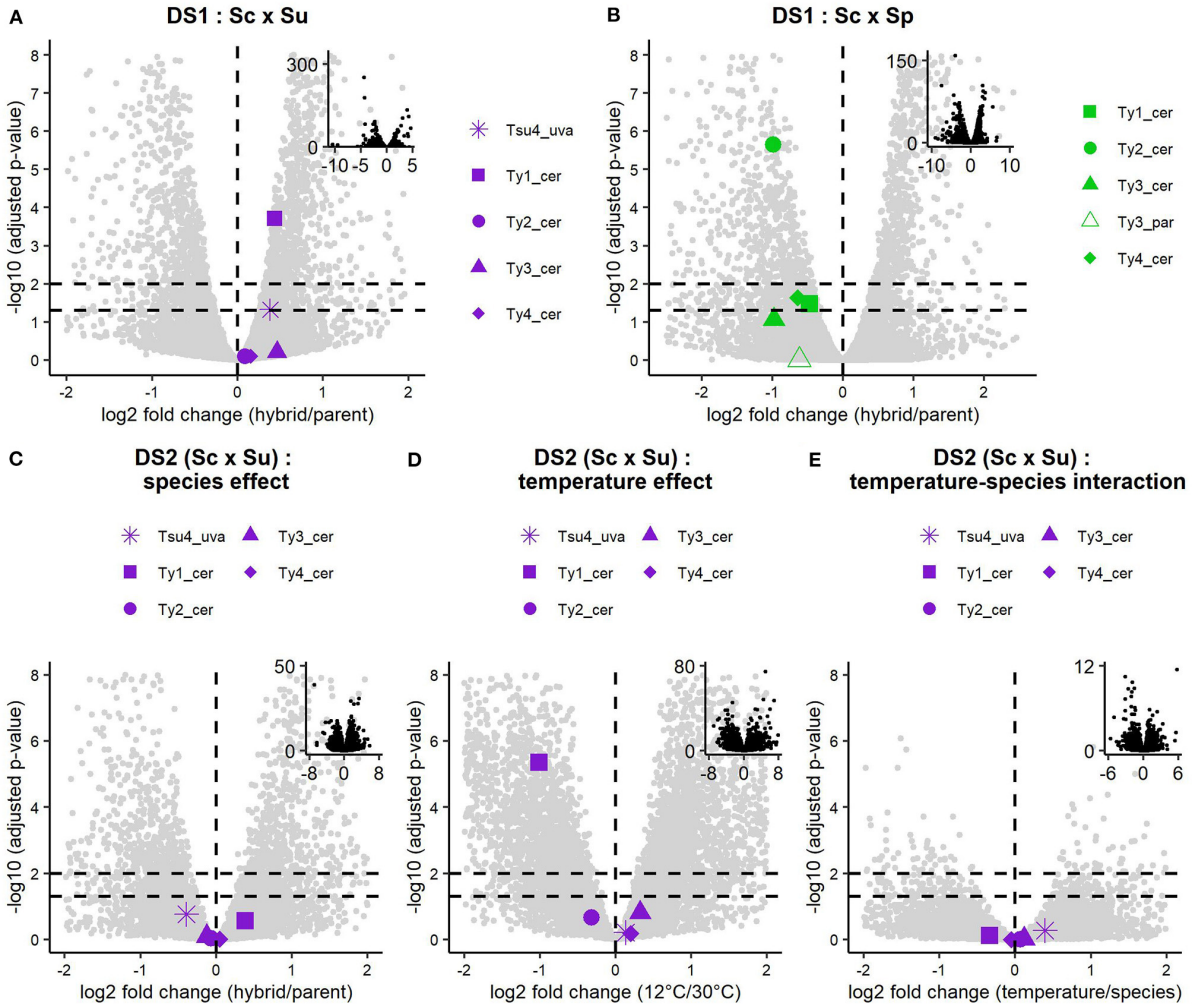
Differential expression analysis results of Ty elements in DS1 and DS2. Negative log_10_ adjusted *p*-value are shown against log_2_ fold change of hybrid vs. parent transcript levels for each active Ty family in DS1 Sc x Su **(A)** and Sc x Sp **(B)**. Results of the multifactor analysis of DS2, including species effect, temperature effect, and temperature-species interaction are respectively shown in **(C–E)**. Non-Ty host genes are shown as background grey dots. The vertical dashed line indicates a log_2_ fold change of 0, while horizontal dashed lines indicate adjusted *p*-values of 0.05 and 0.01. Adjusted *p*-values correspond to Benjamini-Hochberg adjusted *p*-values corrected for multiple tests with the False Discovery Rate (FDR). Insets show the complete data. Ty5_par is absent in **(B)** because no *p*-values could be produced due to low mean normalized counts.

**Figure 4:**
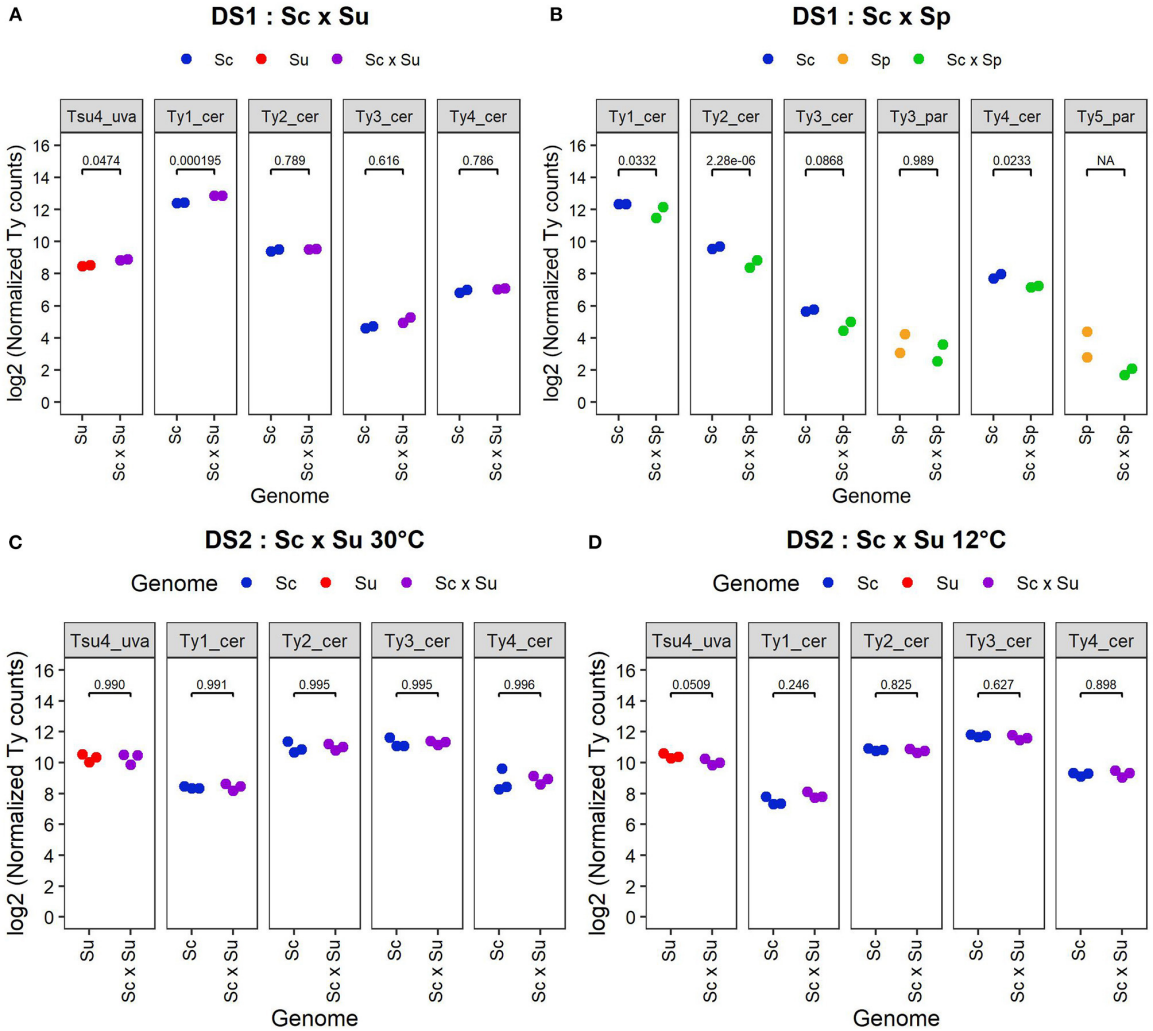
Normalized read counts from DS1 **(A,B)** and DS2 **(C,D)**. Log_2_ of normalized read counts for each active Ty family are shown for *S. cerevisiae* (YHL068) parent, *S. cerevisiae x S. uvarum* hybrid and *S. uvarum* (CBS 7001) parent from DS1 **(A)**; for *S. cerevisiae* (YHL068) parent, *S. cerevisiae x S. paradoxus* hybrid and *S. paradoxus* (CBS432) parent from DS1 **(B)**; for *S. cerevisiae* (YPS128) parent, *S. cerevisiae x S. uvarum* hybrid and *S. uvarum* (UWOPS99-807.1.1) parent from DS2 grown at 30°C **(C)** and 12°C **(D)**. Normalized counts correspond to pseudo normalized counts since the normalized values have been increased by 1 before the log transformation. Note that unlike other Ty families, the analysis of Ty1_cer in Sc x Sp hybrids was done with the exclusion of its GAG region.

**Figure 5:**
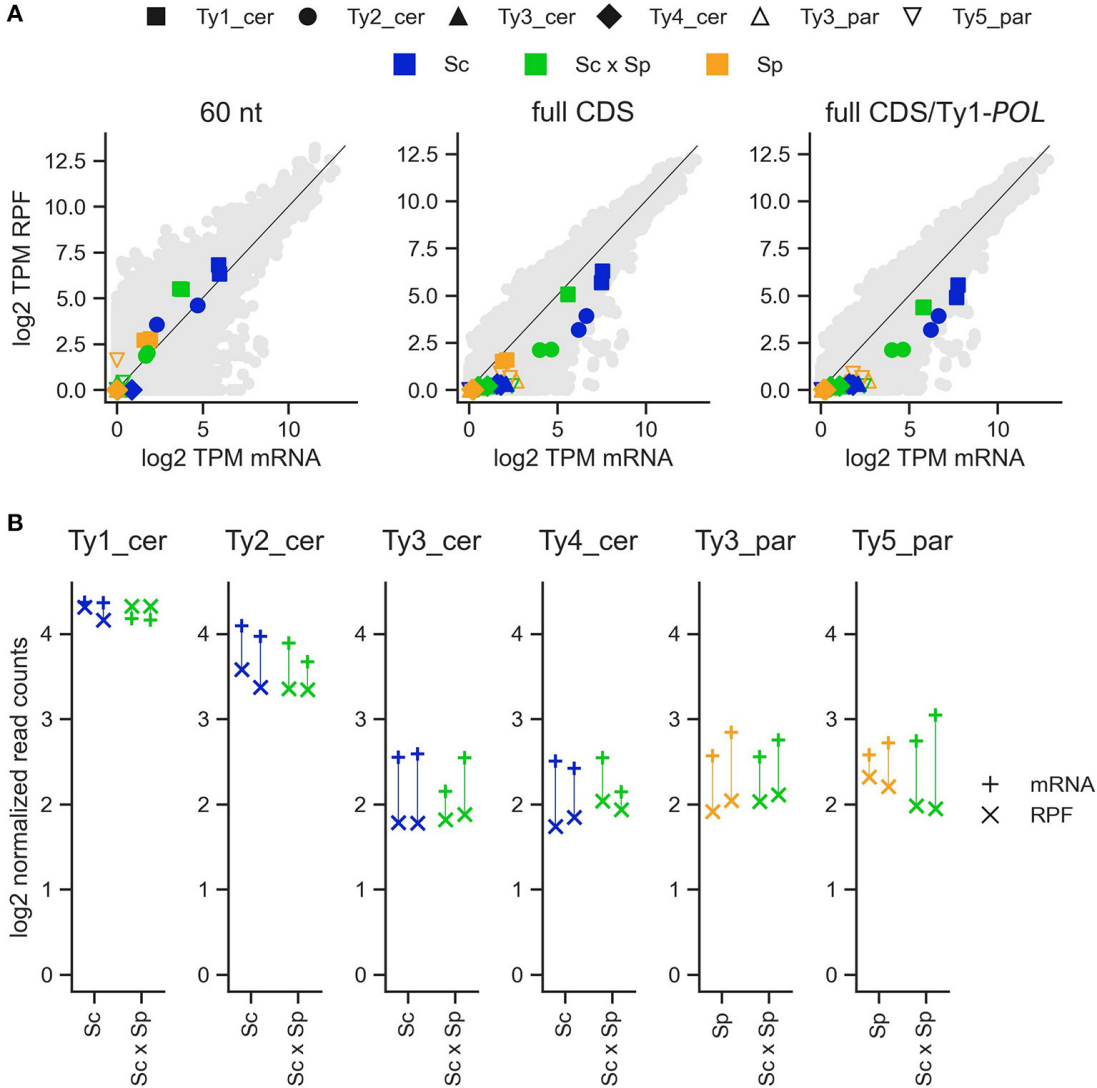
Translation efficiency of Ty families in *S. cerevisiae* × *S. paradoxus* hybrids and their parents. **(A)** Expression level per Ty family for matched replicates of total mRNA and ribosome profiling (RPF) RNA-seq experiments. Host genes are shown as background grey dots. Left: Transcripts Per Million (TPM) for the first 60 nucleotides of each CDS. Center: TPM for the entire length of each CDS. Right: TPM for the entire length of each CDS, but with Ty1_cer in both species truncated to the *POL*-specific region only. **(B)** Normalized read counts for total mRNA and RPF libraries for each Ty family. Matching replicates are joined by vertical lines.

We performed differential expression analysis of the whole transcriptomes of DS1, DS2, and DS3, comparing hybrids and their parents. Analysis of DS1 reveals differences in Ty expression patterns between Sc × Su and Sc × Sp hybrids and their respective parental species (Figures 3A,B, 4A,B). Transcript levels are significantly higher in the Sc x Su hybrid for Tsu4_uva (log2 fold change: 0.383, FDR corrected *p*-value: 0.0474) and Ty1_cer (log2 fold change: 0.433, FDR corrected *p*-value: 1.95 × 10^−4^), whereas other Ty families show similar expression (Figures 3A, 4A). No Ty family is overexpressed in Sc × Sp hybrids (Figures 3B, 4B). Instead, Ty1_cer, Ty2_cer, and Ty4_cer are significantly underexpressed in hybrids, while the other Tys show no significant difference. Since Ty1_cer is subject to asymmetrical non-specific mapping (Figure 2B), we aimed to assess the robustness of our differential expression analysis for this family. Only ~2% of the *S. paradoxus* Ty1 reads are estimated to map on Ty1_cer, so we considered this non-specific mapping negligeable. We manually raised the Ty1_cer read counts by an amount corresponding to the estimated rate of non-specific mapping of *S. cerevisiae* Ty1 reads on Ty1_par (~12%). This analysis showed that, even after our manual count correction, Ty1_cer is still significantly underexpressed in Sc × Sp hybrids. The multi-factor analysis of DS2 reveals that, when grouping samples grown at both 30 and 12°C, there is no Ty family overexpressed in Sc × Su hybrids compared to their parents (Figure 3C). Except for Ty1, temperature does not significantly affect the expression of Ty families (Figure 3D) and there is no significant interaction between species and temperature factors (Figure 3E). We also analysed samples of both temperatures independently (Figures 4C,D). Our results reveal that all the Ty families, either grown at 12 or 30°C, show similar expression in Sc × Su hybrids and in *S. cerevisiae* or in *S. uvarum* (Figures 3C, 4D). Even if Ty1 expression is slightly higher in hybrids, the difference is not significant. Our results show that Ty elements are not more often differentially expressed in hybrids compared to in their parents when they are grown at 12°C. This suggests that Ty regulation remains unaffected by hybridization, even in the low temperature condition. Finally, Sc × Sp hybrids in DS3 show no significant difference in transcript levels for any Ty family (Figure 5B, Supplementary Figure 3).

In the three datasets, the normalized read counts for each Ty family is consistent with the copy number of Ty complete sequences in parental genomes (Figures 2, 5, Supplementary Table 3). Since the total normalized read counts correspond to the sum of the transcript levels of each individual Ty copy within a given family, this result suggests that average transcript levels per copy are fairly consistent across families. Ty3_cer of DS2 is an exception because it is more expressed than Ty2_cer even though the S. *cerevisiae* genome contains fewer copies of Ty3 than Ty2.

In summary, differential expression analysis reveals no sign of systematic genomic shock effect on Ty expression in hybrids. Among the 26 hybrid-parent comparisons, only 2 Ty families show a significant expression increase in hybrids (Ty1_cer and Tsu4_uva in Sc x Su hybrids of DS1).

### Translation Efficiency

**Figure 6:**
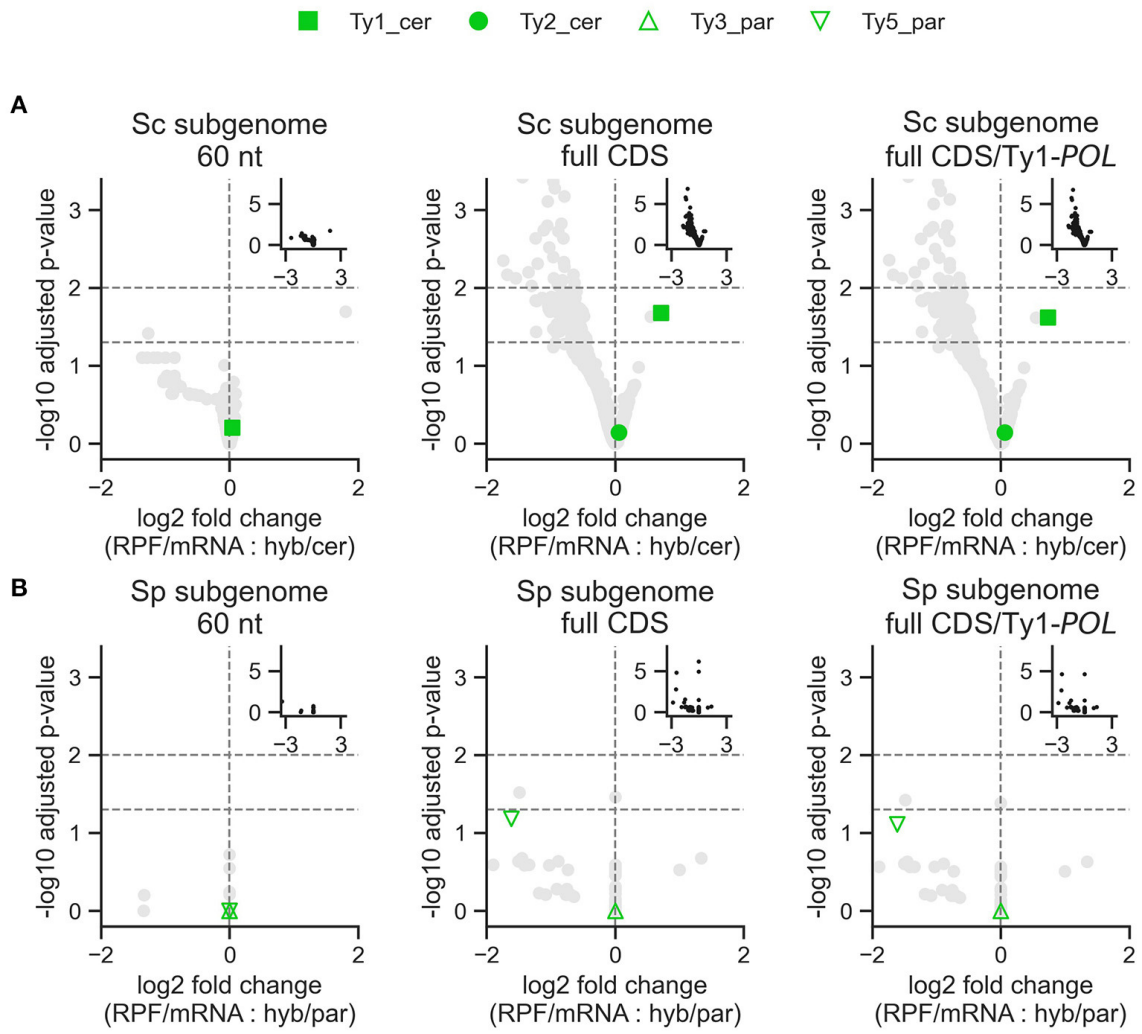
Differential expression analysis of Ty translation efficiency in *S. cerevisiae* x *S. paradoxus* hybrids from DS3. Log_2_ fold change is shown for the two interaction terms in the model: RNA-seq experiment (RPF vs. total mRNA) with the difference between the hybrid and the *S. cerevisiae* **(A)** or *S. paradoxus* **(B)** parent. Positive log_2_ fold change values represent higher translation efficiency in the hybrid compared to the parental species. Insets show the complete data. Ty3_cer and Ty4_cer are absent from the graphs because no *p*-values could be produced.

While Ty families generally show no significant difference in transcript levels, hybridization could modulate Ty expression at other levels. The level immediately downstream of transcription is translation efficiency, which corresponds to the intensity of ribosome occupancy per mRNA molecule. To examine this, we re-analyzed the ribosome profiling data of Sc × Sp hybrids in DS3 (McManus et al., 2014) to test for differences in translation efficiency between hybrids and their parents (Figure 5; Supplementary Figures 1, 3, 4). When measured for the initial 60 nucleotides of the coding sequence (CDS) of each Ty family, none exhibit a significant difference in translation efficiency between the parents and the hybrid (Figure 6). The same analysis on full-length CDS revealed significantly higher translation efficiency in the hybrid for the Ty1 family of *S. cerevisiae*. It also revealed substantial expression of Ty1_cer in the *S. paradoxus* parent as observed in DS1. This signal is consistent with the close relatedness of the *GAG* Ty1 ORF between species (Supplementary Figure 2, Czaja et al., 2020), as its exclusion revealed markedly decreased expression for the *POL*-exclusive region only, particularly for Ty1_cer (Figure 5A). In Ty1, the expression of the full-length *POL* ORF relies on a programmed frameshift that escapes the premature stop codon of the *GAG* ORF. Thus, our results suggest that the regulation of this frameshift in hybrids may lead to a significant increase in translational efficiency.

## Discussion

In this study, we investigated the transcriptional and translational activity of yeast TE families in different genomic contexts and environmental conditions to examine whether they were more expressed in hybrids. We focused on TE transcription and translation levels since yeast retrotransposon activity has mostly been studied by measuring transposition rate in genomes. We performed differential expression analysis on two RNA-seq datasets (DS1, Schraiber et al., 2013; and DS2, Hovhannisyan et al., 2020) and one ribosome profiling dataset (DS3, McManus et al., 2014) of *Saccharomyces* yeast hybrids. To investigate if total expression of Ty families are higher in hybrids than in their parents, we quantified RNA-seq reads mapping on a library of reference sequences comprising every known Ty family. We also evaluated translation efficiency in Sc × Sp hybrids (DS3) by quantifying read mappings from ribosome-protected mRNA fragments.

Overall, our results demonstrate that hybridization generally does not alter TE expression level, with few exceptions of small effect sizes. Among the 26 hybrid-parent total mRNA comparisons, only two Ty families show a significant but small expression increase in hybrids (Ty1_cer and Tsu4_uva in Sc × Su hybrids of DS1). Other Ty families are either not differentially expressed (21/26) or significantly less expressed (3/26) in hybrids. The only overexpressed families were found in the hybrids having the highest parental divergence level, as *S. uvarum* is more distant phylogenetically to *S. cerevisiae* than *S. paradoxus* is (Alsammar and Delneri, 2020). *S. uvarum* is 20 million years divergent from *S. cerevisiae* (Smukowski Heil et al., 2021) whereas *S. paradoxus* and *S. cerevisiae* diverged 5–10 million years ago (Tirosh et al., 2009). This result appears consistent with regulatory divergence increasing with time and causing an accumulation of incompatibilities. Although we would expect to see the impact of a genomic shock at higher frequency in the more divergent species, unlike DS1, our analyses of the same species cross (Sc × Su hybrids) in DS2 revealed no overexpression. The three datasets include different parental strains, growth conditions and RNA-seq library preparation methods. While our analyses included two Sc × Sp hybrids, they correspond to the same parental strains (YHL068 being a close derivative of S288C) and were grown at similar temperatures (25 and 30°C). In contrast, the three Sc × Su hybrids spanned more diversity in terms of parental strains and culture conditions. The higher diversity sampled in Sc × Su hybrids likely increases the odds of observing significant differences in Ty expression. Thus, we found no systematic effect of genomic shock following hybridization on Ty transcriptional activity, and no strong support for a role of regulatory divergence in hybrids.

Our results are consistent with two recent studies showing no transposition rate increase in yeast hybrids (Hénault et al., 2020; Smukowski Heil et al., 2021), as well as with many studies in plants showing no expression changes in hybrids for most TE families (Josefsson et al., 2006; Goebel et al., 2018). Our findings contrast with studies performed in numerous systems such as *Drosophila* (Kelleher et al., 2012; Lopez-Maestre et al., 2017), fish (Dion-Côté et al., 2014; Laporte et al., 2019), and plant (Renaut et al., 2014) showing an overexpression of some TE families in hybrids. Our work also contrasts with studies that found a significantly higher retrotransposon transposition rate in *Drosophila* and mammalian hybrids (O'Neill et al., 1998; Labrador et al., 1999). Patterns of TE expression seems to vary depending on species and types of elements.

TE transcriptional reactivation in diverse fungi has been shown in many stress conditions, including nutrient starvation and host infection stress (Esnault et al., 2019; Fouch et al., 2020). In plants, de-repression of TEs under stress usually impacts TE transcription levels and can increase transpositional activity (Dubin et al., 2018). *S. cerevisiae*'s Ty1 can be transcriptionally activated following adenylic nucleotide depletion (Servant et al., 2012) and have transposition rate 100 times higher at 15°C or 20°C compared to 30°C (Paquin and Williamson, 1984; Garfinkel et al., 2005). In addition to samples grown near the optimal growth temperature of *S. cerevisiae* in the laboratory (25 or 30°C), the datasets we reanalyzed comprised samples grown at a lower temperature (12°C), allowing us to investigate the thermosensitivity of Ty1 (Boeke et al., 1986). Again, we found Ty expression to be the same in hybrids and parents. Sc × Su hybrids include the cryoteolerant species *S. uvarum* (Salvadó et al., 2011), which could mitigate Ty mobilization at low temperatures. Our results are similar to the ones reported by Goebel and collaborators, who found no increase in TE expression in *Arabidopsis* hybrids, even when they were exposed to dehydration stress (Goebel et al., 2018). Our results suggest that changing the environment in which yeast hybridization occurs has a limited impact on retrotransposon activity in hybrids, although we cannot exclude that other conditions or stresses may have an impact.

To extend our analysis beyond transcription, we analyzed a published dataset comprising standard mRNA-seq and RPF sequencing of Sc × Sp artificial hybrids (McManus et al., 2014). Our analyses revealed contrasting results depending on the method employed. Quantification of RPF reads mapping to the first 60 nucleotides downstream of start codons should provide a readout that approximates the level of translation initiation (Ingolia, 2014). Using this metric, no Ty family showed differential translation efficiency in hybrids. However, when considering RPF levels over the whole length of coding sequences, Ty1_cer was translated more efficiently in hybrids. The *POL*-specific section of Ty1's coding sequence appears sufficient to yield this pattern. The *POL* ORF is translated following a programmed frameshift which escapes the stop codon of *GAG* (Curcio et al., 2015). Our results may be explained by a higher frameshifting rate in hybrids, yielding more *POL* translation per initiation event. In *S. cerevisiae*, one factor known to regulate the frequency of frameshifting is the abundance of tRNA-Arg(CCU), which binds the codon right before the frameshift (Kawakami et al., 1993). Alternatively, translation of the *POL*-specific may be slower in hybrids, yielding higher ribosome occupancy levels. Disentangling these hypotheses will require direct quantification of *Gag* and *Gag-Pol* protein abundance in hybrids. Overall, these results suggest that the regulation of Ty expression can be affected by other means than transcription in hybrids.

We see four main limitations to our study. The first one is the challenge to estimate Ty transcription level in some datasets in which sequence coverage of the elements is highly heterogeneous. The fact that an important proportion of the mRNA signal comes from small regions of the Ty sequences could be explained by the presence of alternative polyadenylation sites in Ty sequences. For example, the expression peak at around 800–1,000 bp in Ty1_cer of DS2 corresponds to the 5′ end of the Ty1 internal (Ty1i) transcript, a shorter sense strand RNA encoding Gag protein p22 and p18 (Saha et al., 2015). The peak is in accordance with previously published northern blots reporting Ty alternative transcripts (Fulton et al., 1988; Hug and Feldmann, 1996). Further analysis would be needed to confirm that the different patterns of expression observed in other Ty families also correspond to alternative transcripts. The second limitation is that we quantified transcript abundance at the level of whole families instead of individual elements. In *Saccharomyces*, most full-length Ty elements not yet lost by LTR-LTR recombination are young and highly similar (Carr et al., 2012; Hénault et al., 2020), hindering their discrimination by short read mapping. Individual copies may exhibit wide variation in expression within a Ty family, as illustrated by the 50-fold expression range measured for Ty1 copies in *S. cerevisiae* (Morillon et al., 2002). Thus, we cannot exclude that some Ty copies are overexpressed in hybrids. Whether hybridization alters the variance in transcript levels across individual copies, rather than the mean, remains an open question. The third limitation is that TE mobilization in interspecific hybrids could also be explained by mechanisms that we did not investigate. For instance, a recent study suggests that mitochondrial DNA inheritance from one parent species or the other can affect transposition rate (Smukowski Heil et al., 2021). None of the artificial hybrids reported in the studies we re-analyzed were characterized for mitochondrial DNA inheritance. The final limitation is that the genomic stability of yeast hybrids may depend on the genotypes of the parental strains used (Marsit et al., 2021) and impact the accumulation of TEs (Hénault et al., 2020). Taken individually, our analyses used a single strain per species, offering an incomplete assessment of the intraspecific diversity even if taken together they cover some parental strains of Sc and Su.

The nature of TE repression mechanisms in yeast may provide limited opportunity for the deregulation of TE activity levels in hybrids (Hénault, 2021). TE overexpression has been mostly observed in species regulating TE propagation at the transcriptional level. Mammals and plants mostly repress transposition via epigenetic modifications affecting chromatin accessibility such as DNA methylation and histone modification (Slotkin and Martienssen, 2007). In *Drosophila*, there are two principal small RNA pathways repressing TE mobilization, the piRNA (piwi-interacting RNA) pathway and the siRNA (small interfering RNA) pathway (Merel et al., 2020). Unlike many elements and species, yeast Ty1 elements are self-repressed by a post-translational copy number control (CNC) mechanism that prevents the transposition of an already abundant Ty family (Saha et al., 2015; Czaja et al., 2020). This means that the Ty1 elements from the parental genomes come with their own regulatory mechanisms repressing their own transposition. The fact that Ty1 repression is genome dependent could explain why Ty1 is only overexpressed in some hybrids on DS1. For example, if the strength of self-repression depends on Ty copy number (Garfinkel et al., 2003), variation in Ty copy number among parental strains could determine the strength of the regulation in the resulting hybrids.

In conclusion, our results suggest that Ty transcriptional regulation is quite robust to interspecific hybridization in yeast since we found only a few cases of Ty differential expression. Further studies of TE mobilization incorporating diverse species, TE family repertoires and environmental conditions are needed to assess whether our findings can be generalized to fungal hybridization.

## Data Availability Statement

Publicly available datasets were analyzed in this study. This data can be found on NCBI Sequence Read Archive (SRA). The accession numbers of DS1 (Schraiber et al., 2013) correspond to SRR515220-SRR515231 and SRR835213-SRR835218 (GEO accession number: GSE38875). The accession numbers of DS2 (Hovhannisyan et al., 2020) are SRR10246851-SRR10246868 (Bioproject number: PRJNA576452). The accession number of DS3 (McManus et al., 2014) are SRR948549-SRR948564 (Bioproject number: PRJNA213844).

## Author Contributions

MD, MH, JH, and CRL contributed to the conception and design of the study. MD performed the analysis of DS1 and DS2 and wrote the first draft of the manuscript. MH performed the analysis of DS3. MD, MH, and CRL wrote sections of the manuscript. All authors contributed to manuscript revision, read, and approved the submitted version.

## Conflict of Interest

The authors declare that the research was conducted in the absence of any commercial or financial relationships that could be construed as a potential conflict of interest.

## Publisher's Note

All claims expressed in this article are solely those of the authors and do not necessarily represent those of their affiliated organizations, or those of the publisher, the editors and the reviewers. Any product that may be evaluated in this article, or claim that may be made by its manufacturer, is not guaranteed or endorsed by the publisher.
