# Supplementary material for: Testing the Genomic Shock Hypothesis Using Transposable Element Expression in Yeast Hybrids
Source: Front Fungal Biol. 2021 Aug 23;2:729264. doi: 10.3389/ffunb.2021.729264 (PMC10512236; doi:10.3389/ffunb.2021.729264)
Supplement: Supplementary file 4 [file Data_Sheet_1.pdf]

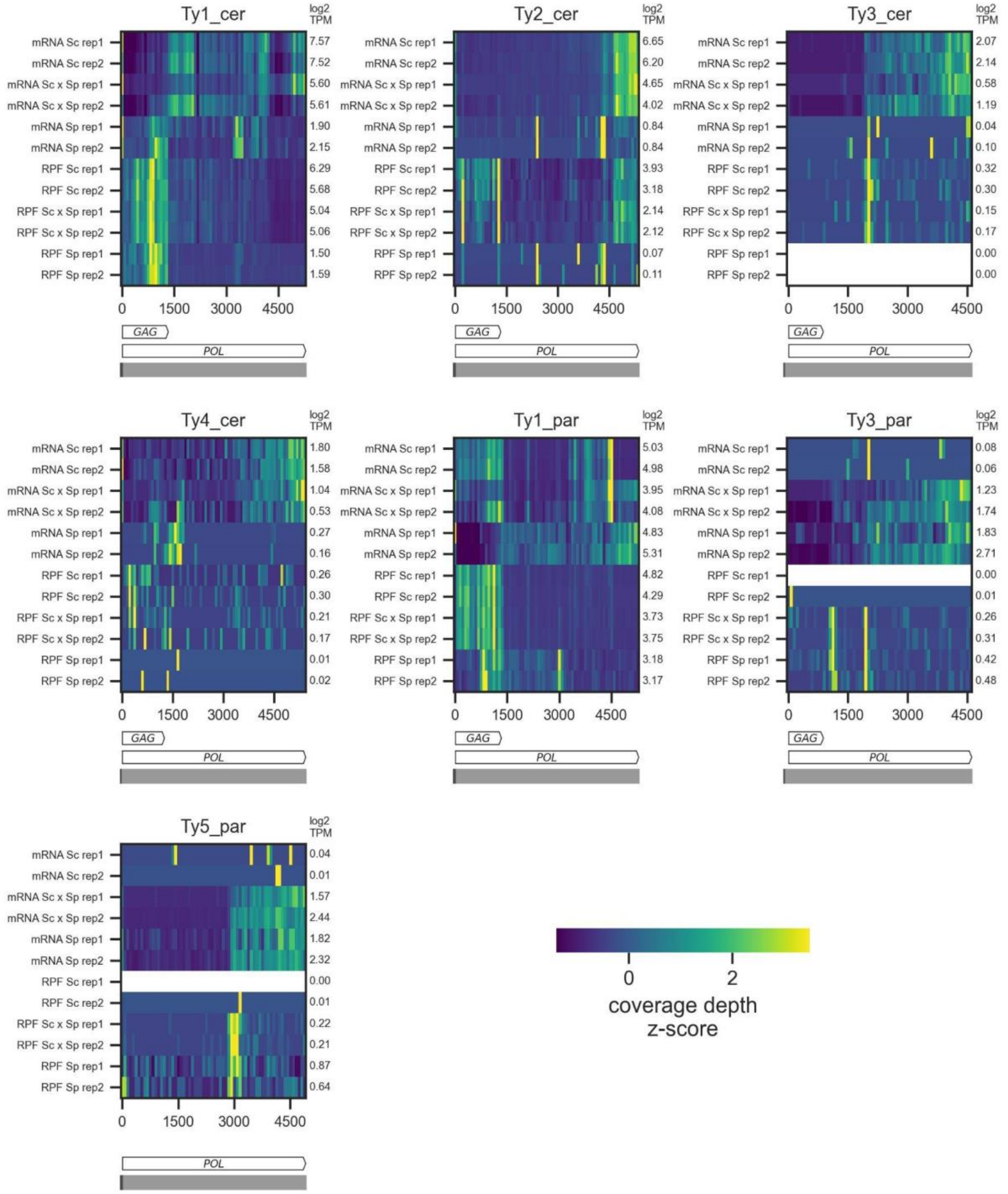


**Supplemental figure 1. Coverage distributions are consistent within Ty families for DS3.** Z-scores of mean coverage depth per 75bp non overlapping windows along Ty reference sequences are shown. Rows show the two replicates for each parental species and hybrids, in both total mRNA sequencing and ribosome profiling (RPF). TPM values of each sample are shown at the right of the heatmaps. Horizontal bars below heatmaps represent the annotation of the reference sequences used for read mapping, with internal sequences and LTRs shown respectively in light and dark grey. Annotations of the POL and GAG ORFs (for Ty1 only) are shown.
