# Supplementary material for: Testing the Genomic Shock Hypothesis Using Transposable Element Expression in Yeast Hybrids
Source: Front Fungal Biol. 2021 Aug 23;2:729264. doi: 10.3389/ffunb.2021.729264 (PMC10512236; doi:10.3389/ffunb.2021.729264)
Supplement: Supplementary file 5 [file Data_Sheet_2.pdf]

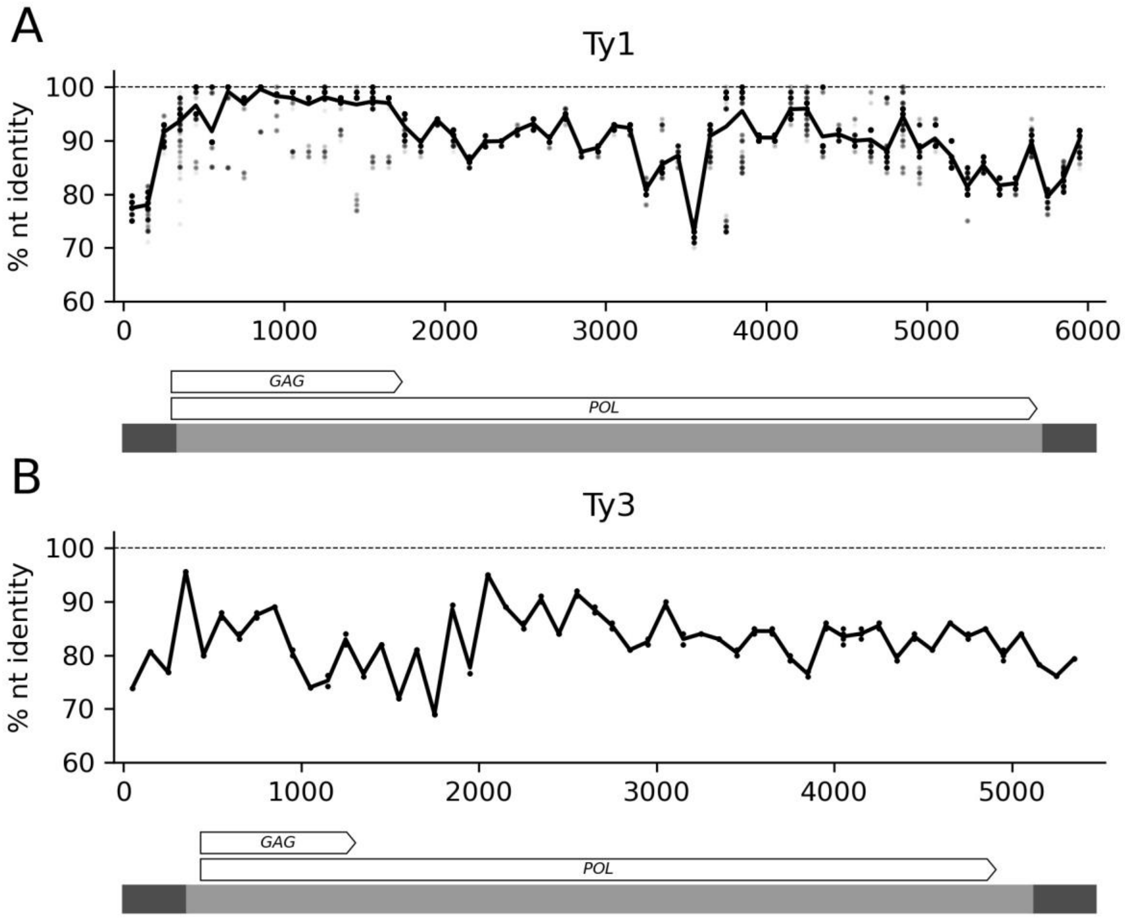

Supplemental figure 2. Average percentage of nucleotide identity between Ty1_cer and Ty1_par **(A)** and between Ty3_cer and Ty3_par **(B)**. Nucleotide identity was computed for non-overlapping 100 bp windows between all possible pairs of full-length sequences extracted from the *S. cerevisiae* S288c genome (40 Ty1 sequences and two Ty3 sequences) and the *S. paradoxus* CBS432 genome (nine Ty1 sequences and two Ty3 sequences; Yue et al. 2017). Points show individual pairwise comparisons, while the solid line shows the average nucleotide identity across comparisons. For Ty1, transparency was added to individual points for enhanced visualization.
