# Supplementary material for: Testing the Genomic Shock Hypothesis Using Transposable Element Expression in Yeast Hybrids
Source: Front Fungal Biol. 2021 Aug 23;2:729264. doi: 10.3389/ffunb.2021.729264 (PMC10512236; doi:10.3389/ffunb.2021.729264)
Supplement: Supplementary file 6 [file Data_Sheet_3.pdf]

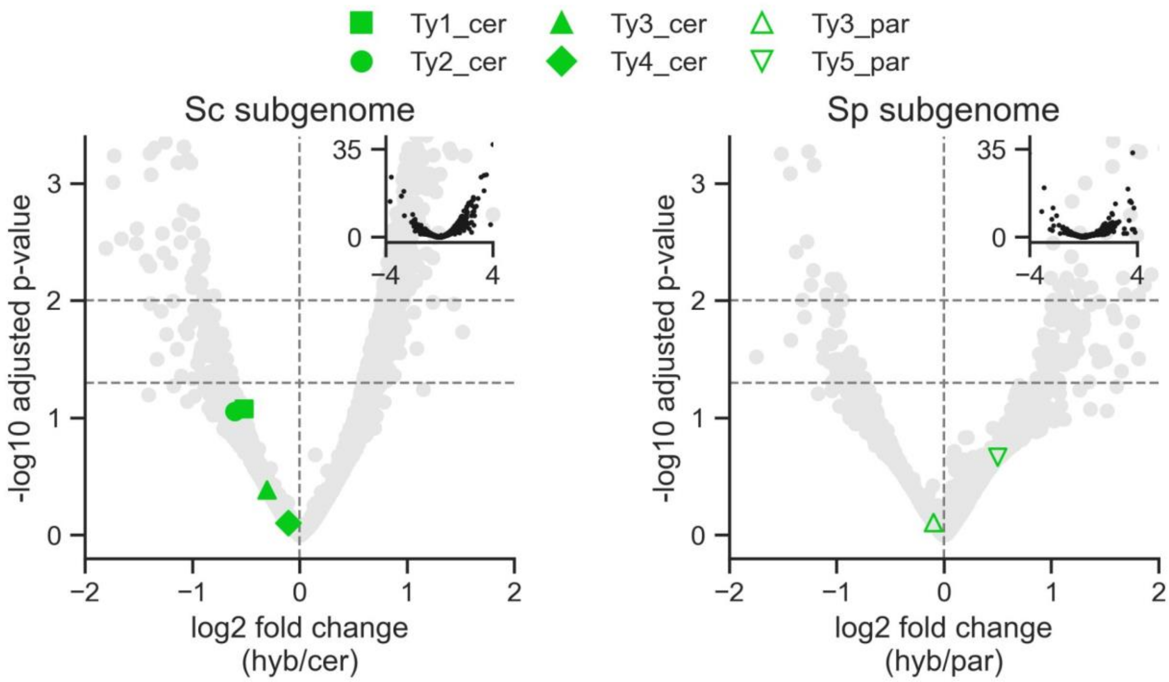

Supplemental figure 3. Differential expression analysis of Ty transcription in *S. cerevisiae* x *S. paradoxus* hybrids from DS3. Log_2 fold change is shown for the difference between the hybrid and the *S. cerevisiae* **(A)** or *S. paradoxus* **(B)** parent. Positive log_2 fold change values represent higher transcript levels in the hybrid compared to the parental species. Insets show the complete data.
