# Supplementary material for: Testing the Genomic Shock Hypothesis Using Transposable Element Expression in Yeast Hybrids
Source: Front Fungal Biol. 2021 Aug 23;2:729264. doi: 10.3389/ffunb.2021.729264 (PMC10512236; doi:10.3389/ffunb.2021.729264)
Supplement: Supplementary file 7 [file Data_Sheet_4.pdf]

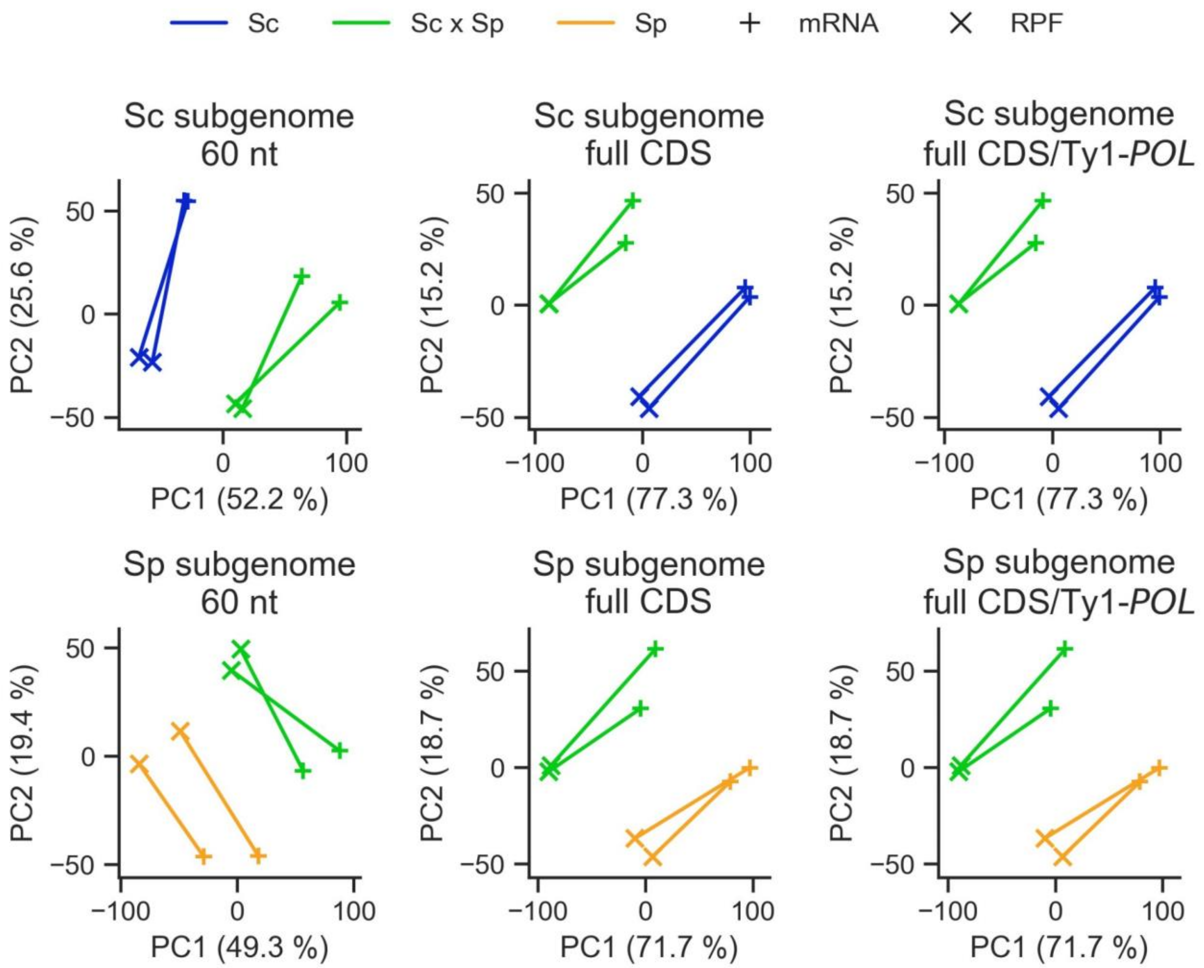

Supplemental figure 4. Principal component analysis on read count data from DS3. Matched mRNA and RPF replicate libraries are connected by solid lines.
